# Multimodal observable cues in mood, anxiety, and borderline personality disorders: a review of reviews to inform explainable AI in mental health

**DOI:** 10.3389/frai.2025.1696448

**Published:** 2025-12-09

**Authors:** Grega Močnik, Ana Rehberger, Žan Smogavc, Izidor Mlakar, Urška Smrke, Sara Močnik

**Affiliations:** 1 Laboratory for Digital Signal Processing, Faculty of Electrical Engineering and Computer Science, University of Maribor, Maribor, Slovenia; 2 Department of Psychology, Faculty of Arts, University of Maribor, Maribor, Slovenia; 3 Unit for Paediatric and Adolescent Psychiatry, Division of Paediatrics, University Medical Centre Maribor, Maribor, Slovenia

**Keywords:** observable cues, mood disorders, anxiety disorders, borderline personality disorder, review, explainable AI

## Abstract

Mental health disorders, such as depression, anxiety, and borderline personality disorder (BPD), are common, often begin early, and can cause profound impairment. Traditional assessments rely heavily on subjective reports and clinical observation, which can be inconsistent and biased. Recent advances in AI offer a promising complement by analyzing objective, observable cues from speech, language, facial expressions, physiological signals, and digital behavior. Explainable AI ensures these patterns remain interpretable and clinically meaningful. A synthesis of 24 recent systematic and scoping reviews shows that depression is linked to self-focused negative language, slowed and monotonous speech, reduced facial expressivity, disrupted sleep and activity, and altered phone or online behavior. Anxiety disorders present with negative language bias, monotone speech with pauses, physiological hyperarousal, and avoidance-related behaviors. BPD exhibits more complex patterns, including impersonal or externally focused language, speech dysregulation, paradoxical facial expressions, autonomic dysregulation, and socially ambivalent behaviors. Some cues, like reduced heart rate variability and flattened speech, appear across conditions, suggesting shared transdiagnostic mechanisms, while BPD’s interpersonal and emotional ambivalence stands out. These findings highlight the potential of observable, digitally measurable cues to complement traditional assessments, enabling earlier detection, ongoing monitoring, and more personalized interventions in psychiatry.

## 1 Introduction

Mental health disorders such as mood disorders (e.g., depression), anxiety disorders and borderline personality disorder (BPD) are among the most prevalent and disabling conditions worldwide. They often begin in adolescence or early adulthood, are highly comorbid, cause serious functional impairment, and carry an elevated mortality risk (Polanczyk et al., 2015; Solmi et al., 2022). Despite growing research, reliable detection and effective treatment of these conditions remain challenging. Psychiatric assessments rely predominantly on subjective methods such as self-report, clinical interviews, and clinician observation, which are variable, and prone to bias (Zimmerman, 2024; Newson et al., 2020). Structured interviews exist but are underutilized, especially in non-specialist settings, potentially missing subclinical or atypical cases (Allsopp et al., 2019). The absence of objective biomarkers further limits diagnostic precision (Pratt and Hall, 2018), compounded by the heterogeneity within diagnostic categories—for example, there are 227 possible ways to meet the symptom criteria for major depressive disorder (Zimmerman et al., 2015).

Artificial intelligence (AI) has advanced rapidly in fields like oncology, radiology, and dermatology, by analyzing standardized, image-based data (Zhou et al., 2025; Hosny et al., 2018; Behara et al., 2024). Psychiatry, in contrast, has been slower to adopt AI due to the subjectivity of symptoms, diagnostic heterogeneity, and ethical concerns surrounding sensitive psychological data (Lee et al., 2021). Nonetheless, AI shows promise for early detection, risk stratification, and personalized mental health care (Baydili et al., 2025).

AI enables the integration of large-scale, multimodal data, including behavioral, linguistic, and physiological signals, to uncover complex, non-linear patterns beyond human perception (Chen X. et al., 2024). For example, AI can predict depression severity from speech and facial expressions (Sadeghi et al., 2024). These tools can detect subtle mental health indicators and improve diagnostic accuracy and timing (Abd-Alrazaq et al., 2023).

To be clinically viable, AI must be interpretable. Explainable AI (XAI) addresses this need by prioritizing transparency and accountability, making algorithmic outputs understandable to both clinicians and patients (Amann et al., 2020).

Observable cues offer a foundation for XAI. These objective behavioral and physiological indicators, captured via smartphones, wearables, or webcams, include speech features, facial expressions, language use, nonverbal behaviors, heart rate variability, and digital activity patterns (Sheikh et al., 2021; Liu et al., 2025; Smrke et al., 2024). Although we use the term “objective” throughout, it refers to the raw data being captured independently of human perceptual bias (unlike self-reports or clinician ratings) (Zimmerman, 2024; Newson et al., 2020). Downstream machine-learning processing (e.g., facial emotion recognition models) can nevertheless introduce algorithmic biases related to training data demographics or representation, (Aquino, 2023; Shahbazi et al., 2023) a point we return to in the limitations section. Multimodal integration of such cues reflect spontaneous, ecologically valid behavior, aligning with the principles of digital phenotyping (Moura et al., 2023; König et al., 2022).

Empirical findings support the clinical relevance of these cues. Remote assessments combining audiovisual and physiological data have demonstrated high accuracy in identifying symptoms of depression and anxiety (Merritt and Zak, 2024; Chen J. et al., 2024). Language-based features, such as excessive self-referential pronouns and negative emotion words, correlate with depression and suicidal ideation (Trifu et al., 2024). Smartphone-derived behavioral metrics including call frequency and screen time, predict mood instability and psychosocial stress (Tng and Yang, 2024; Brodersen et al., 2022).

This review synthesizes findings from scoping reviews, systematic reviews, and meta-analyses on multimodal, objectively measurable cues across mood disorders, anxiety disorders, and BPD. Its unique contribution lies in providing the first synthesis to explicitly map both the clinical feasibility and the passive-sensing potential of these multimodal cues across the three disorder groups—an integration not achieved in prior single-cue or single-disorder reviews. In addition, we examine the methodological, ethical, and explainable-AI implications of using such cues in clinical practice. By combining these perspectives, we highlight the translational potential of validated multimodal digital biomarkers to support transparent AI systems that enable earlier detection, personalized intervention, and ethically responsible psychiatric care.

## 2 Methods

### 2.1 Overview

**Figure 1:**
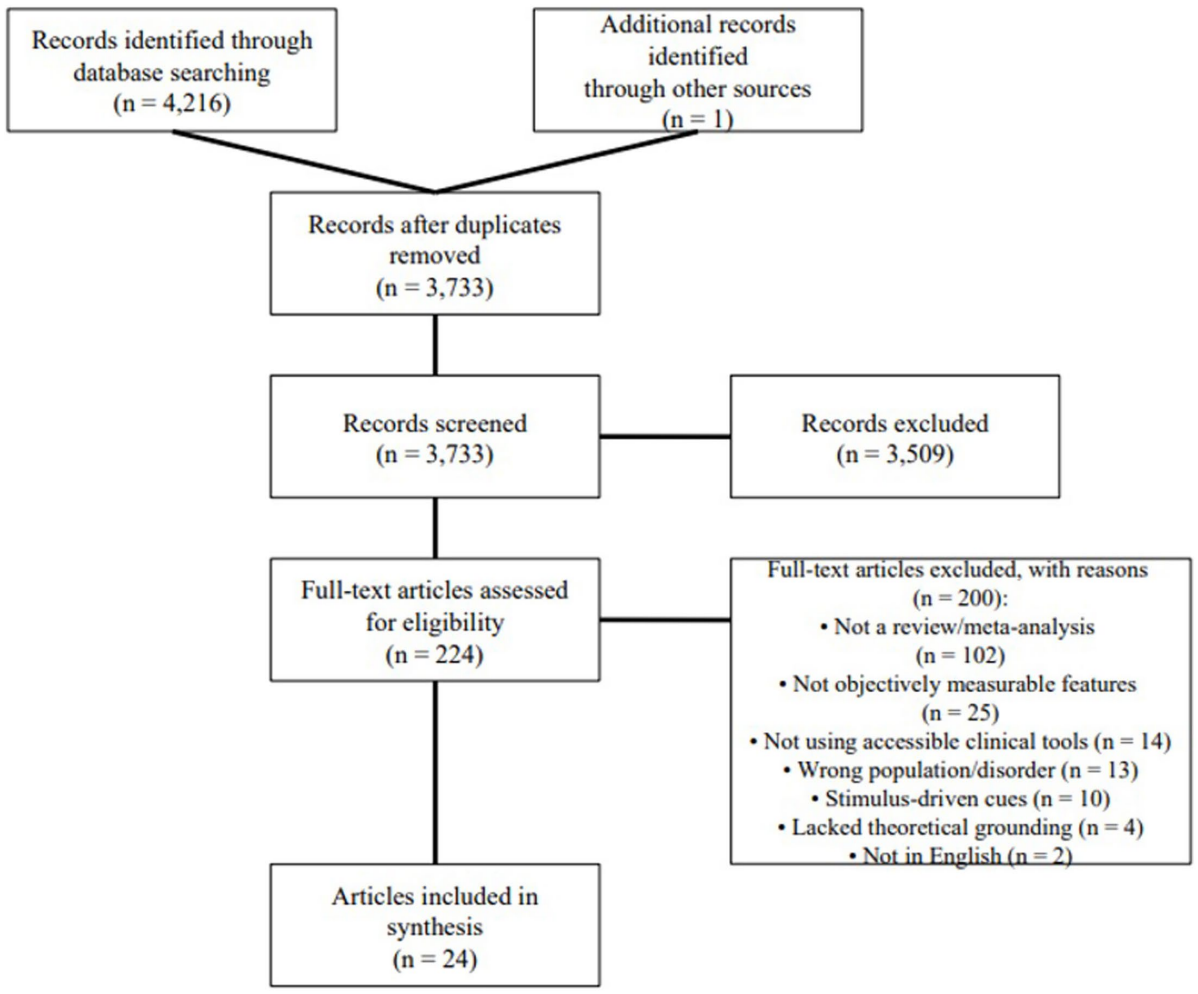
PRISMA flowchart showing the selection process.

We conducted a scoping review following Arksey and O’Malley (2005) and Levac et al. (2010). Accordingly, we: (1) formulated the research questions, (2) identified relevant literature, (3) selected studies for inclusion, (4) charted key data, and (5) synthesized and reported the findings. To ensure a transparent and systematic process, we adhered to the PRISMA-ScR (Preferred Reporting Items for Systematic Reviews and Meta-Analyses extension for Scoping Reviews) guidelines (Tricco et al., 2018), as illustrated in Figure 1.

### 2.2 Identifying the research question

Our investigation was guided by two primary research questions: (RQ1) What observable cues of borderline personality disorder, mood disorders, or anxiety disorders are reported in existing review articles? and (RQ2) Which observable cues are shared across these disorders, and which ones are distinct enough to differentiate among them?

We focused on objectively measurable, spontaneously expressed indicators, including language, speech, facial expressions, nonverbal behavior, physiological signals, and other behavioral metrics. To guide this investigation, we established specific inclusion and exclusion criteria, which are explained in the following section.

### 2.3 Identifying relevant studies

Searches were performed in Web of Science (WoS) and Scopus, the two leading citation bases that include a vast collection of articles from diverse databases such as MEDLINE and ProQuest (Zhu and Liu, 2020). In WoS, we excluded preprint articles and put no constraint on year of publishing. After refining our search to create the most optimal search string, we conducted the main search on December 10, 2024. We used the following final search string in both databases to identify relevant studies: (“borderline personality disorder” OR “emotionally unstable personality disorder” OR “emotional intensity disorder” OR “mood disorder*” OR “affective disorder*” OR “depressive disorder*” OR depression OR MDD OR dysthymi* OR “anxiety disorder*” OR GAD OR PTSD OR C-PTSD OR CPTSD OR “posttraumatic stress disorder” OR “post traumatic stress disorder” OR “panic disorder” OR “social phobi*”) AND (“digital indicator*” OR “physiological indicator*” OR “digital indices” OR “physiological indices” OR “digital biomarker*” OR “physiological biomarker*” OR “digital phenotyp*” OR “digital measure*” OR “physiological measure*” OR “observable cue*” OR “behavio?ral cue*” OR “digital sign” OR “digital signs” OR “digital signal*” OR “physiological marker*” OR “digital marker*” OR feature*) AND (text OR video OR image OR audio OR speech OR language OR paralinguistic OR prosodic OR semantic OR acoustic OR lexical OR facial OR visual OR appearance-based OR vocal OR written OR verbal OR nonverbal OR conversational OR exhalation OR inhalation OR “respiratory rate” OR “breath* amplitude” OR “breath* rate” OR “respiratory pattern” OR “skin temperature” OR “body temperature” OR “skin conductance” OR “electrodermal activity” OR “galvanic skin response” OR “body radiation” OR “sweat gland* activity” OR “heart rate” OR HRV OR “respiratory sinus arrythmia” OR “blood pressure” OR “corrected Qt” OR Qtc OR “blood volume pulse” OR “cardiovascular reactivity” OR “pupillary response” OR “blink rate” OR “pupil dilation” OR pupillometry OR “pupil size variation” OR “eye movement monitoring” OR “saccadic movements” OR “blink frequency”) AND (review OR “meta analysis” OR meta-analysis OR metaanalysis OR “meta-review”) NOT (“autonomic hyperact*” OR Alzheimer* OR Parkinson* OR hyperactivation OR “sympathetic hyperactivity” OR depressor OR “cutaneous depression” OR “respiratory depression” OR “synaptic depression” OR “potentiation/depression” OR dementia OR autism OR autistic OR “neurological disorder” OR stroke OR Huntington* OR paralysis OR mutism OR “multiple sclerosis” OR “cerebral palsy” OR “Down syndrome” OR mouse OR mice OR rat* OR rodent* OR “speech disorder” OR “language disorder” OR “visual impairment”). Our search terms by category are presented in Table 1.

**Table 1:** Terms used in our search strategy by categories.

Category	Relevant mental disorders	Digital indicator	Specific digital indicators	Type of paper	Terms for exclusion
Terms	“Borderline personality disorder” “emotionally unstable personality disorder” “emotional intensity disorder” “mood disorder*” “affective disorder*” “depressive disorder*” depression MDD dysthymi* “anxiety disorder*” GAD PTSD C-PTSD CPTSD “posttraumatic stress disorder” “post traumatic stress disorder” “panic disorder” “social phobi*”	“Digital indicator*” “physiological indicator*” “digital indices” “physiological indices” “digital biomarker*” “physiological biomarker*” “digital phenotyp*” “digital measure*” “physiological measure*” “observable cue*” “behavio?ral cue*” “digital sign” “digital signs” “digital signal*” “physiological marker*” “digital marker*” feature*	Text video image audio speech language paralinguistic prosodic semantic acoustic lexical OR facial visual appearance-based vocal written verbal nonverbal conversational exhalation inhalation “respiratory rate” “breath* amplitude” “breath* rate” “respiratory pattern” “skin temperature” “body temperature” “skin conductance” “electrodermal activity” “galvanic skin response” “body radiation” “sweat gland* activity” “heart rate” HRV “respiratory sinus arrythmia” “blood pressure” “corrected Qt” Qtc “blood volume pulse” “cardiovascular reactivity” “pupillary response” “blink rate” “pupil dilation” pupillometry “pupil size variation” “eye movement monitoring” “saccadic movements” “blink frequency”	Review “meta analysis” meta-analysis metaanalysis “meta-review”	“Autonomic hyperact*” Alzheimer* Parkinson* hyperactivation “sympathetic hyperactivity” depressor “cutaneous depression” “respiratory depression” “synaptic depression” “potentiation/depression” dementia autism autistic “neurological disorder” stroke Huntington* paralysis mutism “multiple sclerosis” “cerebral palsy” “Down syndrome” mouse mice rat* rodent* “speech disorder” “language disorder” “visual impairment”

Specific indicators include physiological indicators.

To be considered for inclusion, articles had to: (1) be a scoping, systematic review, or meta-analysis; (2) focus primarily on objectively measurable observable cues in borderline personality disorder, mood disorders, or anxiety disorders; and (3) be written in English.

Articles were excluded if they: (1) included clinical populations outside the target disorders, (2) did not feature objectively measurable cues, (3) relied predominantly on non-clinically accessible equipment (e.g., MRI, CT, EEG, facial EMG), (4) examined only task- or stimulus-induced responses, or (5) proposed cues without grounding in experimentally tested theory.

Methodological quality was not an exclusion criterion.

### 2.4 Study selection

The main search resulted in 4,216 English-language records (Scopus: 2,970, Web of Science: 1,246) from peer-reviewed articles and conference proceedings. To expand the coverage, a supplementary search within Google Scholar was conducted, adding one additional qualified article. After removing 484 duplicate records, 3,733 unique articles were screened. At title and abstract screening, 224 articles (6.0% of unique records) seemed to satisfy inclusion criteria. Following full-text assessment, only 24 articles (0.6% of unique records) met full inclusion criteria and were included in the final synthesis. This substantial attrition reflects both a deliberately broad search string designed to capture diverse cue types and the application of rigorous, transparently reported exclusion criteria to ensure clinical relevance and feasibility. Consistent with PRISMA-ScR guidelines (Tricco et al., 2018) which make critical appraisal optional for scoping reviews, we did not conduct formal risk-of-bias or quality assessment of the 24 included reviews, as our goal was to map the breadth of the literature and the source articles were already high-level syntheses.

### 2.5 Charting the data

Based on the research questions, we made a structured spreadsheet to guide data extraction from the included reviews. The following information was recorded: (1) authors, (2) year of publication, (3) type of article, (4) number of primary studies covered, (5) reported inclusion and exclusion criteria, (6) disorders relevant to our scope, (7) observed cues categorized by type and disorder, (8) methods of observation, and (9) limitations noted by the authors. Data were extracted independently by four researchers (GM, AR, ŽS, and SM) and refined through an iterative process during the review.

### 2.6 Collating, summarizing, and reporting results

We adhered to the goal of scoping reviews, i.e., the charting of current evidence and its descriptive representation (Arksey and O’Malley, 2005). After extraction of data by GM, AR, ŽS, and SM, data were subsequently subject to thematic analysis by authors GM, AR, and SM. Cue categories were predefined and covered: (1) language, (2) speech, (3) facial expressions, (4) other nonverbal communication, (5) physiological measures, and (6) behavioral cues. Charting and summarizing of findings were checked by three researchers (IM, US, and SM).

## 3 Results

### 3.1 Characteristics of reviewed studies

Of the 24 included articles, 17 were systematic reviews, 6 scoping reviews, and 1 without a specified type. The earliest review was published in 2018 (Rohani et al., 2018) and the most recent in 2024 (Smrke et al., 2024; Choi et al., 2024; Khoo et al., 2024; Ko et al., 2024; Liu et al., 2024; Močnik et al., 2024; Shin and Bae, 2024; Zierer et al., 2024). On average, reviews included 60 articles (SD = 50), with the highest number being 184 (Khoo et al., 2024) and the lowest 9 (Smrke et al., 2021). With respect to mental disorders, 21 articles investigated observable cues related to mood disorders (20 on depression and 7 on bipolar disorder), 10 addressed anxiety disorders, and one examined BPD. The observable cues studied were diverse and included language (10 studies), speech (10 studies), facial expressions (5 studies), and physiological cues (10 studies) measured using various methods such as ECG, wristbands, EEG, EMG, Ag/AgCl electrodes, and respiration sensors. Other domains of interest were nonverbal communication [1 study; (Pampouchidou et al., 2019)], phone use (11 studies), mobility and activity cues (14 studies), sleep (5 studies), and online activity (3 studies). In many studies, wearable hardware (e.g., mobile phones, wristbands) and software (e.g., GPS, calls and texts tracking, Wi-Fi, bluetooth, cellular network, accelerometer, actigraphy, audio recording) was used to collect observable cues. The full characteristics of the reviewed studies are presented in Table 2.

**Table 2:** Summary of the articles included in the review.

Author(s) and year	Type of paper	N	Relevant disorders	Observed cues for mood disorders	Observed cues for anxiety disorders	Observed cues for BPD	Methods of observation
Bhadra and Kumar (2022)	Systematic review	135	Depression	Motor activity	/	/	/
Bufano et al. (2023)	Systematic review	29	Bipolar and depressive disorder	Mobility and activity, social rhythm	/	/	Smartphone sensors and wearable wristbands
Chesnut et al. (2021)	Scoping review	13	Depression, anxiety	Physiological cues	Physiological cues	/	HRV, skin conductance
Choi et al. (2024)	Systematic review	40	Anxiety, mild depression	Language, sleep, phone use, physical activity, mobility, motor activity	Language, sleep, phone use, physical activity, mobility, motor activity	/	GPS, microphone, light sensor, accelerometer, phone use, incoming and outgoing calls/messages/emails, bluetooth, Wi-Fi, keyboard (typing patterns and muscle activity, app use, gyroscope)
Dhelim et al. (2023)	Systematic review	120	Anxiety, depression	Language and behavioral (app use)	Behavioral cues (phone use)	/	Social media analysis
Fraccaro et al. (2019)	Systematic review	16	Bipolar disorder	Mobility and activity	/	/	GPS, Wi-Fi, Bluetooth, or GSM cellular network
Hilty et al. (2021)	Scoping review	92	Depressive and bipolar disorder and anxiety disorders	Mobility and activity, phone use	Physiological	/	Smartphones, wristbands, smartwatches, holters; most common sensors being accelerometer, phone use, GPS, microphone, actigraph, ECG
Khoo et al. (2024)	Systematic review	184	Depression, anxiety	Language and behavioral (online activity, physical mobility, phone use)	Behavioral cues (online activity)	/	/
Ko et al. (2024)	Systematic review and meta-analysis	22	Anxiety	/	Physiological, facial muscle activity	/	Facial recognition, EEG
Liu et al. (2024)	Systematic review	114	Depression	Language, speech, facial expressions	/	/	/
Low et al. (2020)	Systematic review	127	Depression, bipolar disorder, anxiety, PTSD	Speech (acoustic features)	Speech (acoustic features)	/	Automated speech feature extraction
Maatoug et al. (2022)	Systematic review	45	Depressive and bipolar disorder	Language, speech, physiological, and behavioral cues (mobility and activity, phone use)	/	/	Speech analysis, GPS, actigraphy, phone use
Močnik et al. (2024)	Scoping review	24	BPD	/	/	Language use, speech patterns, facial expressions, nonverbal communication, physiological cues	Various methods, including ECG, Ag/AgCl electrodes, EMG, videotaping, audio recording, and coding systems
Pampouchidou et al. (2019)	Systematic review	‘over 60’	Depression	Facial expressions and nonverbal cues	/	/	Facial recognition, nonverbal communication recognition
Rohani et al. (2018)	Systematic review	46	Depression	Phone use, mobility and activity, sleep	/	/	Mobile phone or other wearable device
Saccaro et al. (2021)	Systematic review	62	Depression, bipolar disorder	Language, speech, physiological, mobility and activity, sleep, phone use	/	/	GPS position, phone apps, wearable sensors, audio/video feature extraction
Shahimi et al. (2022)	Systematic review	12	Depression, anxiety	Physiological cues	Physiological cues	/	Blood pressure variability (time domain variability or frequency domain)
Sheikh et al. (2021)	*NA*	73	Depression, bipolar disorder, anxiety, PTSD	Mobility and activity	Physiological cues, sleep	/	Wearable devices, mobile phones, ambient sensors
Shin and Bae (2024)	Scoping review	14	Depression	Speech	/	/	Microphone, recording predetermined speech, capturing intentional spontaneous speech
Shin and Bae (2023)	Systematic review	31	Depression	Phone use, mobility and activity (physical activity)	/	/	GPS and GSM cellular network, accelerometer, Wi-Fi, cell tower, mobile phone tower triangulation, Wi-Fi network, and bluetooth-based location data
Smrke et al. (2024)	Scoping review	33	Anxiety disorder	/	Language, speech, physiological, mobility and activity	/	Smartphone data, social media data, audio recordings, respiration sensor, wristband, actigraphy, GPS
Smrke et al. (2021)	Scoping meta-review	9	Depression	Language, speech, facial expression	/	/	/
Zarate et al. (2022)	Systematic review	118	Depression	Speech, physical activity, sleep, phone use	/	/	Mobile sensing
Zierer et al. (2024)	Systematic review	27	Depression	Language, speech, physiological cues, physical activity, app use, sleep	/	/	Sensors, smartphones or wearable device, location

N, number of primary studies included.

### 3.2 Observed cues for mood disorders

This section provides an overview of the findings of 21 review studies that examined observable or physiologically measurable cues of mood disorders, particularly depressive and bipolar disorder.

#### 3.2.1 Cues related to language use

Regarding language use cues in mood disorders, the most consistent finding is that people with depression use more first-person language and pronouns (Khoo et al., 2024; Zierer et al., 2024; Smrke et al., 2021; Maatoug et al., 2022). They also express negative emotions more frequently (Khoo et al., 2024; Zierer et al., 2024; Smrke et al., 2021; Maatoug et al., 2022; Dhelim et al., 2023). In addition, reviews report that people with depression use fewer positive emotion words (Smrke et al., 2021) and use more death-related words (Choi et al., 2024; Zierer et al., 2024). One study found that third-person pronouns such as “others” are also used more often (Khoo et al., 2024). In terms of language structure, depressed patients tend to use more ruminative language (Zierer et al., 2024; Smrke et al., 2021), and more passive forms such as auxiliaries (Zierer et al., 2024). Their language is often unclear, repetitive, incoherent (Liu et al., 2024), with reduced complexity [i.e., fewer complex sentences, less adverbial clauses; (Smrke et al., 2021)]. We have also found that depressed individuals tend to talk or write less, particularly on social media (Choi et al., 2024; Smrke et al., 2021), although other studies report mixed results (Dhelim et al., 2023). Saccaro et al. (2021) found that depression is associated with a higher number of characters in both incoming and outgoing text messages, whereas manic states were associated with fewer characters in incoming texts. Specific lexical markers of depression include words related to pain (Smrke et al., 2021; Dhelim et al., 2023), treatment (“side effects” and “therapy”), absolutist expressions (e.g., “always,” “never”), past-oriented terms (e.g., “learned,” “remember”) (Smrke et al., 2021), words indicating daily life stress and calls for support (Dhelim et al., 2023), expressions of low self-esteem and pessimism (Liu et al., 2024), and reward-related words (Choi et al., 2024). More specific markers include” hurt,” “tears,”” alone,” “hate,” “sleep,” and “worry” (Smrke et al., 2021), as well as “depressed,” “hopeless,” and “worthless” (Khoo et al., 2024).

#### 3.2.2 Cues related to speech

In terms of speech, the most consistent findings across studies are that depressed individuals speak less overall (Shin and Bae, 2024; Zierer et al., 2024; Zarate et al., 2022). They tend to produce shorter phrases and engage less in verbal communication (Smrke et al., 2021). Depression is also marked by a slower speech rate (Liu et al., 2024; Smrke et al., 2021), longer pauses during speech initiation and conversation (Smrke et al., 2021; Maatoug et al., 2022; Low et al., 2020), and increased response latency (Liu et al., 2024; Maatoug et al., 2022). A negative correlation between pitch and loudness and the severity of depression has been shown (Liu et al., 2024). Several studies consistently report reduced variation in pitch and loudness and reduced F0 range, resulting in monotonous speech (Liu et al., 2024; Smrke et al., 2021; Low et al., 2020). Relatedly, less frequently reported cues linked to depression include lack of linguistic stress (Smrke et al., 2021). Jitter and shimmer are also commonly observed (Smrke et al., 2021; Low et al., 2020). In comparisons to healthy controls, manic states have been linked to increases in pitch, F1, F2, and F4 values (Saccaro et al., 2021; Low et al., 2020), manic patients’ vocal tracts have also been shown to produce more distinct or pronounced spectral patterns (Saccaro et al., 2021). Depression was also associated with unclear articulation (Liu et al., 2024; Smrke et al., 2021). Acoustic analyses reveal alternated formant frequencies in F2; decreases in depression (Smrke et al., 2021) and increases in mania (Saccaro et al., 2021).

#### 3.2.3 Cues related to facial expressions

Studies have consistently found that depression is associated with reduced facial expressivity (Liu et al., 2024; Smrke et al., 2021; Pampouchidou et al., 2019), particularly with respect to positive emotions (Smrke et al., 2021). Relatedly, reduced activation on the zygomaticus muscle (activates during smiling) and fewer smiles were observed in depressed individuals (Liu et al., 2024; Smrke et al., 2021; Pampouchidou et al., 2019). Depression is also linked to reduced variability and intensity of facial expressions. Depressed individuals more frequently display sad, negative, neutral expressions, and frowns (Smrke et al., 2021), which are linked to downward-angled mouth corners (for sadness) and extended activity on the corrugator muscle [for frowns; (Smrke et al., 2021; Pampouchidou et al., 2019)]. Reduced saccadic eye movements and increased visual fixation have also been observed in depressed individuals (Smrke et al., 2021), along with limited eye contact, which is often shorter or evasive. Glances tend to be lower in both frequency and duration (Liu et al., 2024; Smrke et al., 2021; Pampouchidou et al., 2019) and a greater tendency toward toward downward gaze has been reported (Liu et al., 2024; Smrke et al., 2021). Other characteristics include more frequent lip pressing (Smrke et al., 2021).

#### 3.2.4 Other cues of nonverbal communication

Only one review has included studies that investigate cues of nonverbal communication (Pampouchidou et al., 2019). They found that shaking and/or fidgeting behavior, self-adaptors, and foot tapping, slumped posture, limp and uniform body posture, reduced and slowed arm and hand movements have been considered as signs of depression.

#### 3.2.5 Cues related to physiological measurements

Studies report that depressed individuals show lower heart rate variability (HRV) across time and frequency domains, as well as reduced inter-beat intervals (Zierer et al., 2024; Maatoug et al., 2022; Chesnut et al., 2021), while some studies found a positive relation between HRV and depressive symptom severity (Saccaro et al., 2021). Depressed patients tend to exhibit higher LF/HF ratios, coupled with decreased high-frequency HRV in bipolar disorder (Zierer et al., 2024; Maatoug et al., 2022). HRV was found to be increased in manic states compared to both depressive and euthymic states (Saccaro et al., 2021). Regarding blood pressure, one review reported that individuals with depressive symptoms exhibit higher average systolic and diastolic blood pressure (Shahimi et al., 2022). Furthermore, increased blood pressure variability has been observed in patients with depressive symptoms (Shahimi et al., 2022). Several studies also highlight body temperature anomalies in depression. Findings include decreased temperature, decreased temperature amplitude, lower skin temperature amplitude and less stable skin temperature rhythms, and night-time increases in temperature in depressed individuals (Maatoug et al., 2022). Less frequently reported are findings on skin conductance: depressed individuals exhibit lower tonic skin conductance (Chesnut et al., 2021).

#### 3.2.6 Other behavioral cues

##### 3.2.6.1 Mobility and activity

Depression has consistently been associated with reduced mobility patterns. Depressed individuals tend to visit fewer locations, make fewer exits from home, show lower variance in distance travelled, and visit fewer new places. They also spend more time at home, have a reduced mobility radius, and show lower entropy (a measure of how evenly they spend time across clusters of places; low entropy = time spent in visited places is not evenly distributed) and location variance (Rohani et al., 2018; Choi et al., 2024; Khoo et al., 2024; Zierer et al., 2024; Saccaro et al., 2021; Bufano et al., 2023; Hilty et al., 2021; Shin and Bae, 2023). In contrast, studies report that adolescents with depression travelled longer distances compared to healthy controls (Khoo et al., 2024) and that depression was not associated with staying more time at home (Hilty et al., 2021). For mania, increased mobility and greater travel activity have been reported (Bufano et al., 2023; Hilty et al., 2021), while one study found that distance travelled was negatively associated with mania and the number of cell tower changes to be positively associated with mania but negatively with depression (Fraccaro et al., 2019). Sheikh et al. (2021) have also found mixed findings on association between bipolar mania and distance travelled. In individuals at risk for bipolar disorder, locomotor amplitude index was found to predict future risk (Saccaro et al., 2021). Increased geospatial activity has also been linked to improvements in depression scores (Sheikh et al., 2021).

Depression was consistently associated with reduced physical activity, including fewer daily steps, lower exercise levels, more sedentary behavior, less motion variability, and reduced fine motor movements (Rohani et al., 2018; Choi et al., 2024; Khoo et al., 2024; Zierer et al., 2024; Maatoug et al., 2022; Zarate et al., 2022; Hilty et al., 2021; Bhadra and Kumar, 2022). Some studies also show later activity onset, midday activity peaks, and reduced evening activity in depressed individuals (Maatoug et al., 2022; Saccaro et al., 2021; Hilty et al., 2021). Depression has further been linked to lower nearby Bluetooth device count, lower variance, and less periodicity (Maatoug et al., 2022; Shin and Bae, 2023). Decreased physical activity was also found in bipolar patients (Saccaro et al., 2021).

##### 3.2.6.2 Sleep and circadian rhythm

In depression, disruptions in sleep and circadian patterns are widely observed. Depressed individuals commonly show reduced daytime but increased nighttime activity, irregular circadian rhythms (Rohani et al., 2018; Zierer et al., 2024), delayed sleep phases, greater night-to-night variability, insomnia, poor sleep quality, and increased exposure to light at bedtime (Choi et al., 2024; Zarate et al., 2022). Importantly, sleep architecture and motor activity during sleep show substantial heterogeneity: some individuals demonstrate markedly reduced nocturnal motor activity consistent with hypersomnia, whereas others display increased nighttime movement and sleep fragmentation, reflecting insomnia and psychomotor restlessness (Rohani et al., 2018; Choi et al., 2024; Zierer et al., 2024; Zarate et al., 2022). In bipolar disorder, patients demonstrate shorter sleep duration, more interruptions, and greater variability in day-to-day sleep time compared to healthy controls (Saccaro et al., 2021).

##### 3.2.6.3 Phone use

Depression has been associated with greater overall smartphone use, more variable use patterns, higher nighttime and evening activity, higher screen unlock frequency, longer screen-on times, and shorter unlock durations (Rohani et al., 2018; Choi et al., 2024; Khoo et al., 2024; Zierer et al., 2024; Hilty et al., 2021). In bipolar disorder, screen-on duration was longer in manic and mixed states compared to euthymia (Saccaro et al., 2021). Positive correlations between normalized entropy of screen status and depression have also been described (Maatoug et al., 2022).

Depression is associated with fewer and shorter calls, fewer incoming and answered calls, fewer outgoing calls per day, and fewer contacts saved (Khoo et al., 2024; Zierer et al., 2024; Maatoug et al., 2022; Saccaro et al., 2021; Zarate et al., 2022; Hilty et al., 2021). Longer outgoing call durations have been linked to depression (Choi et al., 2024), however (Rohani et al., 2018) reported low agreement of studies on association between depression and call duration and call frequency. Depressed participants also call fewer individual people than controls (Zierer et al., 2024). In mania, individuals tend to make longer calls and receive more incoming calls (Maatoug et al., 2022; Saccaro et al., 2021; Hilty et al., 2021).

Depressed individuals send more text messages and open messaging apps less frequently (Zierer et al., 2024), but receive fewer messages (Khoo et al., 2024). Other studies found that depressed individuals portrayed reduced SMS frequency (Maatoug et al., 2022; Zarate et al., 2022). Rohani et al. (2018) on the other hand found low agreement among studies regarding the link between received text messages and depression. Same was found with emotionally unstable students who exhibited higher texting frequency (Choi et al., 2024). Manic states, in contrast, are associated with an increase in messages sent (Hilty et al., 2021) compared to depressed states (Saccaro et al., 2021).

##### 3.2.6.4 Social media and online activity

Depressed individuals tend to post more frequently at midnight, but their posts receive less engagement in the form of likes or retweets (Khoo et al., 2024). In addition, posting activity has been negatively correlated with depressive symptoms, and depressed individuals tend to have fewer Facebook friends, while a marginally positive correlation has been found with Instagram use (Khoo et al., 2024; Dhelim et al., 2023). They also disclose less personal information, are more likely to modify images, and are less likely to share location information (Khoo et al., 2024). More broadly, depressed individuals used social applications more frequently and for a longer duration (Khoo et al., 2024). Zierer et al. (2024) also found that using social applications in the evening is a predictor of depressive symptoms.

### 3.3 Observed cues for anxiety disorders

This section synthesizes findings from 10 recent systematic and scoping reviews examining observable or physiologically measurable cues in individuals with anxiety disorders, including generalized anxiety disorder (GAD), panic disorder, social anxiety disorder (SAD), post-traumatic stress disorder (PTSD), and related conditions.

#### 3.3.1 Cues related to language use

Language use in individuals with anxiety disorders revealed several patterns. Most consistently, individuals with social anxiety displayed a negativity bias in language, with increased use of negative emotion words, fewer positive emotion words, and a higher proportion of death-related terms (Smrke et al., 2024). Ambient speech analysis linked GAD to the frequent use of reward-related words and SAD to vision-related terms, potentially indicating attentional biases toward goals and environmental surveillance (Choi et al., 2024).

#### 3.3.2 Cues related to speech

Speech patterns in PTSD and social anxiety showed consistent paralinguistic alterations. PTSD patients exhibited longer pauses, slower speech rates, and lower vocal intensity, especially during trauma recall - features reflective of emotional suppression and cognitive overload (Low et al., 2020). A flatter prosody and monotone delivery were also associated with higher anxiety levels, pointing to disrupted affective modulation in speech (Smrke et al., 2024).

#### 3.3.3 Other cues of nonverbal communication

Across the reviewed literature, no consistent anxiety-specific patterns were reported regarding gaze behavior, gestures, posture, or nonverbal synchrony.

#### 3.3.4 Cues related to physiological measurements

Physiological dysregulation emerged as the most consistent observable cue across anxiety disorders. Reduced HRV and lower respiratory sinus arrhythmia (RSA) were reported in children with anxiety disorders, indicating diminished parasympathetic tone (Ko et al., 2024). Elevated 24-h blood pressure and increased blood pressure variability were associated with GAD (Shahimi et al., 2022). Shortened inhalation/exhalation cycles and irregular respiratory rhythms were also markers of heightened anxiety (Smrke et al., 2024). Consistent with this, significantly reduced time-domain and high-frequency HRV were observed in anxiety populations, reflecting vagal withdrawal (Chesnut et al., 2021). PTSD was associated with elevated electrodermal activity and higher resting heart rate, indicative of increased sympathetic arousal (Hilty et al., 2021).

#### 3.3.5 Other behavioral cues

Several studies linked behavioral patterns with anxiety. Movement data revealed that higher mobility diversity and frequent exits from home predicted lower social anxiety, while restricted transitions (e.g., from education to supermarkets) predicted higher levels (Smrke et al., 2024). App usage patterns showed that anxious individuals spent more time on passive media, gaming, and health-related apps, and behaviors like maintaining distance from virtual characters were linked to SAD Khoo et al., 2024. Increased digital activity during late hours was observed among anxious users (Dhelim et al., 2023). Socially anxious individuals demonstrated reduced movement diversity, avoidance of darkness, increased phone use, and a pattern of evening calls and irregular walking (Choi et al., 2024). Greater time spent at home and avoidance of religious venues correlated with higher SAD (Hilty et al., 2021), and socially anxious students were less engaged in public or leisure activities. PTSD patients showed more nighttime activity and fragmented sleep patterns (Sheikh et al., 2021).

### 3.4 Observed cues for borderline personality disorder

The scoping review conducted by our research group in 2024 remains the only systematic effort to date that specifically examines observable, AI-detectable cues in individuals with BPD. The review categorized observable behaviors and physiological signals into six predefined domains: language use, speech characteristics, facial expressions, nonverbal communication, physiological measures, and other behavioral cues (Močnik et al., 2024).

#### 3.4.1 Cues related to language use

Language use in BPD revealed a distinct stylistic and thematic pattern. Most notably, individuals with BPD exhibited a preference for impersonal and externalized expressions, with increased use of third-person singular pronouns (e.g., “they”) and future tense constructions. Their speech frequently contained negations, intensifiers, conjunctions, and nonfluencies such as “so,” “I mean,” or “because.” Thematic analysis highlighted frequent references to social processes, powerlessness, and biomedical narratives, though some texts emphasized identity, acceptance, and individuality. Notably, a negative emotional tone was more common in written content, while interviews revealed more neutral affect. No significant differences were observed in sentence complexity or semantic richness when compared to controls.

#### 3.4.2 Cues related to speech

Speech in BPD was characterized by elevated pause frequency and a slower speech rate, suggesting conversational dysregulation. Dialogue dynamics showed distinctive coupling between vocal-acoustic features, such as decibels and fundamental frequency, and emotional expressions, particularly adjectives and interjections conveying anger or disgust. Interestingly, the direction of acoustic-emotion correlations (e.g., anger vs. loudness or pitch) differed from those in healthy individuals, suggesting altered affective mapping in speech.

#### 3.4.3 Cues related to facial expressions

Facial expressivity in BPD patients fell into two clusters. One cluster exhibited high expressivity with intense negative emotions like anger, contempt, and disgust, which co-occurred with social smiles – indicating mixed or conflicting emotional signaling. The other cluster showed less intense negative affect but retained affiliative expressions. Compared to controls, individuals with BPD expressed less sadness but more disgust and contempt, suggesting a preference for externalizing emotional states. Overall, emotional signaling in BPD was complex, often blending hostile and affiliative cues. Behavioral markers included diminished affiliative gestures such as smiling and nodding, which typically support social cohesion. Interestingly, individuals with BPD also exhibited persistent social smiles—possibly signaling an attempt to maintain interpersonal engagement despite internal distress. Interactional behavior often reflected ambivalence, with simultaneous cues of withdrawal and social approach, indicating internal conflict in social contexts.

#### 3.4.4 Other cues of nonverbal communication

Nonverbal behavior in BPD revealed atypical social signaling. Patients often led the rhythm of interaction through pseudosynchrony, a form of nonverbal synchrony with conversational partners. Affiliative gestures such as eyebrow raises and head tilts were less frequent, indicating impaired prosocial communication. Flight behaviors, signaling withdrawal, were observed in both BPD and control participants, implying they may be general rather than disorder-specific markers.

#### 3.4.5 Cues related to physiological measurements

Physiological markers in BPD demonstrated signs of autonomic dysregulation. While heart rate (HR) findings were inconsistent, reduced RSA and HRV were consistently linked to symptom severity. Elevated systolic and diastolic blood pressure was observed at rest, along with prolonged QTcd intervals, suggesting altered cardiac repolarization. Sympathetic arousal was evidenced by increased skin conductance levels (SCL), while lower orbicularis oculi EMG activity suggested blunted facial muscle responsiveness.

## 4 Discussion

This review brings together 24 systematic and scoping reviews published between 2018 and 2024 that explored observable, digitally measurable cues across three major psychiatric conditions: mood disorders (depression and bipolar disorder), anxiety disorders, and BPD (Smrke et al., 2024; Rohani et al., 2018; Choi et al., 2024; Khoo et al., 2024; Ko et al., 2024; Liu et al., 2024; Močnik et al., 2024; Shin and Bae, 2024; Zierer et al., 2024). Most studies focused on depression, fewer on anxiety, and only one specifically on BPD (Močnik et al., 2024). Observable cues spanned language and speech, facial expressions, nonverbal behaviors, physiological signals, mobility, sleep, phone use, and online activity (Choi et al., 2024; Khoo et al., 2024; Pampouchidou et al., 2019).

In mood disorders, depression consistently presented with self-focused, negatively valenced language, frequent use of first-person pronouns, death-related words, ruminative and passive expressions, reduced linguistic complexity, and lower social media activity (Choi et al., 2024; Khoo et al., 2024; Liu et al., 2024; Zierer et al., 2024; Smrke et al., 2021). Speech patterns included reduced verbal output, slower rate, longer pauses, monotonous pitch, and increased jitter and shimmer (Liu et al., 2024; Smrke et al., 2021; Maatoug et al., 2022; Low et al., 2020). Facial expressions were blunted, with fewer smiles, reduced zygomaticus activation, increased frowning, and downward gaze (Liu et al., 2024; Smrke et al., 2021; Pampouchidou et al., 2019). Physiological changes involved reduced HRV, altered blood pressure, and disrupted temperature rhythms (Zierer et al., 2024; Maatoug et al., 2022; Chesnut et al., 2021; Shahimi et al., 2022). Behaviorally, depressive states were marked by reduced mobility, lower physical activity, irregular circadian rhythms, disrupted sleep, and changes in phone and online activity (Rohani et al., 2018; Choi et al., 2024; Khoo et al., 2024; Zierer et al., 2024; Saccaro et al., 2021; Bufano et al., 2023; Hilty et al., 2021; Shin and Bae, 2023). In contrast, manic episodes in bipolar disorder showed increased mobility, travel distance, phone activity, and more variable speech (Saccaro et al., 2021; Bufano et al., 2023; Hilty et al., 2021).

Anxiety disorders displayed a distinct pattern. Language reflected a negativity bias, heightened attentional vigilance, and increased use of reward- or vision-related words in GAD and SAD (Smrke et al., 2024; Choi et al., 2024). Speech was slower, monotone, and punctuated by longer pauses, particularly during trauma recall in PTSD (Smrke et al., 2024; Low et al., 2020). Physiological markers consistently included reduced HRV and RSA, sympathetic overactivation, irregular respiratory cycles, and elevated blood pressure (Ko et al., 2024; Chesnut et al., 2021; Shahimi et al., 2022; Hilty et al., 2021). Behaviorally, anxiety was associated with avoidance of public spaces, restricted transitions, increased nighttime activity, and heavier use of passive digital media (Sheikh et al., 2021; Choi et al., 2024; Khoo et al., 2024; Dhelim et al., 2023).

BPD, based on the single scoping review, showed unique and often ambivalent cues (Močnik et al., 2024). Language featured impersonal pronouns, externalized references, negations, intensifiers, and conversational nonfluencies. Speech was slower with more frequent pauses and an altered mapping between acoustic features and expressed emotions. Facial expressions were paradoxical, combining anger, contempt, disgust, and social smiles. Nonverbal communication showed reduced affiliative gestures and atypical pseudosynchrony. Physiologically, BPD was associated with reduced HRV and RSA, elevated blood pressure, prolonged QTc intervals, and increased skin conductance. Behaviorally, individuals demonstrated ambivalent social approach, diminished prosocial gestures, and persistent social smiles despite internal distress.

Across disorders, depression and anxiety share an internalizing profile, whereas BPD is characterized by emotional ambivalence and dysregulation across linguistic, expressive, physiological, and social domains. Shared features like reduced HRV and monotone speech suggest transdiagnostic pathways. Overall, cues in mood and anxiety disorders align with clinical models, while BPD findings provide empirical support for theories of emotional instability and interpersonal dysregulation.

Objective cues can complement traditional assessments, support early detection, track symptom fluctuations, and guide personalized interventions. Clinical integration requires careful attention to privacy, ethics, and standardization (Močnik et al., 2024; Bhadra and Kumar, 2022). Future research should emphasize longitudinal, multimodal studies in diverse populations, especially in BPD where data remain limited.

### 4.1 Ecologically valid multimodal assessment, digital phenotyping, and small-data machine learning

A critical future direction is the development of ecologically valid, real-world multimodal systems that capture cue dynamics in daily life. A recently proposed trimodal framework integrating ecological momentary assessment, physiological measurements, and speech emotion recognition to model emotion dynamics longitudinally presents a promising blueprint for dynamic multimodal digital phenotyping in mental health (Wang et al., 2025).

This approach aligns closely with the broader concept of digital phenotyping, which seeks to redefine psychiatric assessment through continuous, passive, data-driven monitoring in everyday life (Oudin et al., 2023).

Finally, individualized modeling faces the “small-data problem.” Few-shot, transfer-learning, and small-data machine learning techniques are therefore essential to achieve clinically useful personalized models in psychiatry (Koppe et al., 2021).

### 4.2 Study limitations

Several limitations should be noted. First, the evidence is uneven across disorders: most studies have focused on depression and bipolar disorder, with fewer addressing anxiety, and only one review specifically examining BPD (Smrke et al., 2024; Khoo et al., 2024; Močnik et al., 2024). Second, methodological differences across the included reviews make synthesis challenging, as studies varied in how they collected data (e.g., wearable devices, phone tracking, EEG, EMG), the parameters they measured, and the analytic approaches they used (Choi et al., 2024; Zierer et al., 2024; Pampouchidou et al., 2019). Third, many studies were cross-sectional, which limits our ability to draw causal conclusions or understand how observable cues change over time (Saccaro et al., 2021; Hilty et al., 2021). Fourth, the specificity of the markers is limited because some features overlap across disorders - for example, reduced HRV and monotonous speech appear in depression, anxiety, and BPD (Močnik et al., 2024; Chesnut et al., 2021). Finally, most studies focused on adults, which restricts how well the findings can be applied to children, adolescents, or culturally diverse populations (Khoo et al., 2024; Ko et al., 2024).

In addition, we did not conduct a formal critical appraisal of the 24 included reviews. While permissible within the PRISMA-ScR framework (Tricco et al., 2018), the absence of quality assessment remains a limitation; future updates may incorporate methodological weighting. Most source reviews also did not systematically address key confounders, including psychotropic medication, somatic comorbidity, age, substance use, and physical fitness, which are known to influence physiological and behavioral cues (Croatto et al., 2023). Medication effects on HRV represent a particularly important source of reduced disorder-specificity (Quintana et al., 2016). Lastly, although described as “objective,” many digitally measurable cues (e.g., facial emotion recognition, speech-based affect classification) depend on machine-learning models that may inherit demographic or cultural biases from training datasets. These biases warrant caution when interpreting findings and considering clinical deployment.

### 4.3 Clinical implications

Validated multimodal cues offer clear translational value across psychiatry. They can complement traditional assessments by providing digitally measurable insights into emotional, behavioral, and physiological states (Choi et al., 2024; Močnik et al., 2024). In mood disorders, shifts in mobility, sleep, or phone activity may signal emerging depressive or manic episodes and support longitudinal monitoring (Khoo et al., 2024; Hilty et al., 2021). In anxiety disorders, reduced HRV and sympathetic overactivation can indicate heightened arousal and maladaptive coping (Smrke et al., 2024; Ko et al., 2024). For BPD, indicators of ambivalent social signaling, emotional dysregulation, and physiological hyperarousal may enrich clinical interviews, inform risk, and support treatment planning (Močnik et al., 2024). Integrating language, speech, facial expressions, physiological data, and digital behavior enables more personalized interventions and earlier detection while monitoring treatment response. Implementation requires careful attention to privacy, data security, ethical standards, and patient consent (Močnik et al., 2024; Welzel et al., 2025). Ultimately, multimodal digital cues may help differentiate overlapping symptoms and refine individualized care across psychiatric disorders (Sahili et al., 2024).

## 5 Conclusion

Observable, digitally measurable cues provide objective, clinically relevant insights across psychiatric disorders. Depression and anxiety share overlapping internalizing patterns, whereas BPD is distinguished by emotional ambivalence, altered speech-emotion relationships, and paradoxical expressions. These markers can enhance assessment, monitor symptom trajectories, and guide early, individualized interventions. To advance clinical utility, future research should prioritize longitudinal, multimodal studies across diverse populations, improve marker specificity, and validate predictive models. Collectively, these efforts support a more precise, data-informed approach to psychiatric care that complements traditional evaluation and tailors interventions to individual patient profiles.

## Data Availability

The original contributions presented in the study are included in the article/supplementary material, further inquiries can be directed to the corresponding author.
